# Анализ эпидемиологических показателей заболеваний паращитовидных желез в Чувашской республике на основе данных регистра

**DOI:** 10.14341/probl13663

**Published:** 2026-07-22

**Authors:** Г. Р. Айзетуллова, И. В. Слепцов, В. Ф. Осипов, В. С. Балтримас, А. С. Иванова

**Affiliations:** Республиканский клинический госпиталь для ветеранов войн; Чувашский государственный университет имени И.Н. Ульянов Россия; Republican Clinical Hospital for War Veterans; Chuvash State University named after I.N. Ulyanova Russian Federation; Санкт-Петербургский государственный университет Россия; St. Petersburg State University Russian Federation; Чувашский государственный университет имени И.Н. Ульянов Россия; Chuvash State University named after I.N. Ulyanova Russian Federation

**Keywords:** заболевания паращитовидных желез, первичный гиперпаратиреоз, эпидемиология, регистр, распространенность, parathyroid diseases, primary hyperparathyroidism, epidemiology, register, prevalence

## Abstract

**АКТУАЛЬНОСТЬ:**

АКТУАЛЬНОСТЬ. Первичный гиперпаратиреоз представляет собой одно из наиболее распространенных эндокринных заболеваний, характеризующееся нарушением кальций-фосфорного обмена вследствие гиперсекреции паратиреоидного гормона.

**ЦЕЛЬ:**

ЦЕЛЬ. Оценка эпидемиологической ситуации с заболеваниями паращитовидных желез в Чувашской Республике на основе данных Всероссийского регистра пациентов с первичным гиперпаратиреозом. Проанализировать данные пациентов с первичным гиперпаратиреозом в рамках ретроспективного когортного исследования с 2014 по 2025 гг. на клинической базе БУ ЧР «Республиканский клинический госпиталь для ветеранов войн» Минздрава Чувашии.

**МАТЕРИАЛЫ И МЕТОДЫ:**

МАТЕРИАЛЫ И МЕТОДЫ. Проанализированы данные по Чувашской Республике из Всероссийского регистра пациентов с первичным гиперпаратиреозом. В ходе ретроспективного когортного исследования (2014–2025 гг.) была проанализирована когорта пациентов, состоящих на диспансерном учете в БУ ЧР «Республиканский клинический госпиталь для ветеранов войн» Минздрава Чувашии. Для статистической обработки данных применялся описательный метод с расчетом абсолютных и относительных величин (процентных соотношений).

**РЕЗУЛЬТАТЫ:**

РЕЗУЛЬТАТЫ. Установлено, что распространенность патологии паращитовидных желез в регионе составляет 73,4 случая на 100 000 населения. Выявлены ключевые эпидемиологические особенности: выраженное преобладание женщин (88,9%) старшей возрастной группы, неравномерное географическое распределение с концентрацией случаев в урбанизированных территориях (Чебоксары, Новочебоксарск), а также значительный рост выявляемости в последние годы. Полученные данные свидетельствуют о влиянии как организационных факторов (доступность специализированной помощи), так и возможных экологических рисков (дисбаланс микроэлементов в водных источниках) на распространенность патологии.

**ЗАКЛЮЧЕНИЕ:**

ЗАКЛЮЧЕНИЕ. Внедренный Всероссийский регистр доказал свою эффективность для мониторинга заболеваемости, планирования медицинской помощи и проведения научных исследований, что определяет перспективы его дальнейшего развития для оптимизации системы оказания эндокринологической помощи в регионе.

## Abstract (en)

**RELEVANCE:**

RELEVANCE. Primary hyperparathyroidism is one of the most common endocrine diseases characterized by impaired calcium-phosphorus metabolism due to hypersecretion of parathyroid hormone.

**GOAL:**

GOAL. Assessment of the epidemiological situation with parathyroid gland diseases in the Chuvash Republic based on data from the All-Russian Registry of Patients with primary hyperparathyroidism. To analyze the data of patients with primary hyperparathyroidism in a retrospective cohort study from 2014 to 2025 at the clinical base of the Republican Clinical Hospital for War Veterans of the Ministry of Health of Chuvashia.

**MATERIALS AND METHODS:**

MATERIALS AND METHODS. The data on the Chuvash Republic from the All-Russian Registry of patients with primary hyperparathyroidism are analyzed. In the course of a retrospective cohort study (2014–2025), a cohort of patients registered at the Republican Clinical Hospital for War Veterans of the Ministry of Health of Chuvashia was analyzed. For statistical data processing, a descriptive method was used with the calculation of absolute and relative values (percentages).

**RESULTS:**

RESULTS. It was found that the prevalence of parathyroid gland pathology in the region is 73.4 cases per 100,000 population. Key epidemiological features were identified: a pronounced predominance of women (88.9%) in the older age group, an uneven geographical distribution with a concentration of cases in urbanized areas (Cheboksary, Novocheboksarsk), as well as a significant increase in detection in recent years. The data obtained indicate the influence of both organizational factors (availability of specialized care) and possible environmental risks (imbalance of trace elements in water sources) on the prevalence of pathology.

**CONCLUSION:**

CONCLUSION. The implemented electronic registry has proven its effectiveness for monitoring morbidity, planning medical care and conducting scientific research, which determines the prospects for its further development to optimize the system of endocrinological care in the region.

## Введение

Околощитовидные железы (ОЩЖ) — ключевые регуляторы минерального обмена в эндокринной системе, поддерживающие стабильный уровень кальция в крови засчет секреции паратиреоидного гормона (ПТГ) [1]. Нарушение этой функции приводит к развитию заболеваний. Так, избыток ПТГ при опухолевой трансформации вызывает первичный гиперпаратиреоз, а его недостаток приводит к гипопаратиреозу. Эти состояния, которые часто выявляются случайно при исследовании кальциевого обмена в рамках диагностики других патологий, оказывают системное влияние, негативно воздействуя не только на фосфорно-кальциевый обмен, но и на состояние сердечно-сосудистой системы, почек и костной ткани [2]. Первичный гиперпаратиреоз (ПГПТ) — это эндокринное заболевание, вызванное гиперфункцией одной или нескольких паращитовидных желез [3]. Одиночная доброкачественная аденома паращитовидной железы является наиболее распространенным проявлением [4]. ПГПТ является одним из наиболее распространенных эндокринных заболеваний (встречается у 0,3% населения в целом и у 1–3% женщин в постменопаузе), уступая по частоте только сахарному диабету и патологии щитовидной железы [5].

Биохимическими признаками заболевания традиционно являются гиперкальциемия и повышенный уровень ПТГ, однако недавно описанная нормокальциемическая форма ПГПТ, характеризующаяся нормальным уровнем общего и ионизированного кальция в сыворотке крови на фоне явно повышенного уровня паратиреоидного гормона при исключении иных причин его повышения, расширила клинический фенотип заболевания [6][7].

В последние годы широкое внедрение автоматизированного биохимического скрининга, позволяющего измерять концентрацию кальция в сыворотке крови, привело не только к увеличению количества диагностированных случаев гипопаратиреоза, но и изменило его клинический спектр, превратив заболевание из состояния с ярко выраженными симптомами в менее симптоматичное [8]. ПГПТ претерпел изменения в клинической картине и теперь обычно диагностируется на бессимптомной стадии [9].

Широко признано, что паратиреоидэктомия (ПТЭ) является методом выбора для всех пациентов с симптоматическим заболеванием и для пациентов с бессимптомным заболеванием, соответствующих хирургическим критериям. Преимущества оперативного лечения заключаются в нормализации фосфорно-кальциевого обмена и устранении ассоциированных с гиперкальциемией симптомов, значимом улучшении состояния костной ткани и почек, сердечно-сосудистой системы и положительных изменениях когнитивных функций у абсолютного большинства прооперированных пациентов [10]. Медикаментозная терапия с применением фармакологических препаратов является альтернативой для пациентов с бессимптомным ПГПТ, которые соответствуют критериям для проведения операции, но не могут или не хотят подвергаться ПЭ [3].

По данным Всероссийского регистра первичного ГПТ, в 2021 г. было учтено 4400 пациентов из 79 регионов РФ [1]. В международном контексте Швеция, Дания и США ведут национальные регистры, насчитывающие 8–10 тыс. случаев каждая [11][12][13].

Для эффективного планирования как хирургической, так и консервативной тактики ведения пациентов с заболеваниями паращитовидных желез необходима надежная эпидемиологическая основа. Отсутствие достоверных эпидемиологических данных об этой патологии в Чувашской Республике подчеркивает важность информирования и обучения медицинских работников для расширения охвата и повышения полноты заполнения регистра. Анализ заболеваемости и распространенности ПГПТ в регионе позволит выявить возможные кластеры случаев заболевания, оценить влияние экологических, генетических и социально-экономических факторов на его развитие.

## Цель исследования

Оценить распространенность заболеваний паращитовидных желез в Чувашской Республике с использованием данных Всероссийского регистра пациентов с первичным гиперпаратиреозом. Проанализировать данные пациентов с первичным гиперпаратиреозом в рамках ретроспективного когортного исследования с 2014 по 2025 гг. на клинической базе БУ ЧР «Республиканский клинический госпиталь для ветеранов войн» Минздрава Чувашии.

## Материалы и методы исследования

Проведена оценка распространенности заболеваний паращитовидных желез в Чувашской Республике с использованием данных Всероссийского регистра пациентов с первичным гиперпаратиреозом. В период с 2014 по 2025 гг. на клинической базе БУ ЧР «Республиканский клинический госпиталь для ветеранов войн» Минздрава Чувашии была сформирована и проанализирована когорта пациентов с первичным гиперпаратиреозом (ПГПТ) в рамках ретроспективного когортного исследования. Учреждение функционирует как многопрофильный республиканский медицинский центр, оказывающий специализированную помощь как льготным категориям граждан, так и населению из всех районов региона в целом. В его структуре есть хирургическое отделение, профилирующееся на лечении патологии эндокринной системы.

Диагностические возможности при подозрении на ПГПТ в Чувашской Республике включают верификацию диагноза с помощью лабораторных методов (исследование кальций-фосфорного обмена и уровня ПТГ) и последующую топическую диагностику с применением инструментальных методов: УЗИ, сцинтиграфия паращитовидных желез иКТ шеи и средостения.

В ходе исследования для первичного анализа клинико-эпидемиологических данных был применен описательный метод статистического анализа. Этот подход позволил систематизировать и обобщить собранную информацию, представив ее в виде абсолютных и относительных показателей. Для оценки структуры и распределения признаков (таких как пол, форма заболевания, географическая принадлежность) рассчитывались абсолютные значения и процентные соотношения.

## Результаты исследования

Регистр, основанный в Чувашской Республике, показывает положительную динамику пополнения количества пациентов, которая коррелирует с двумя факторами: экстенсивным развитием онлайн-платформы за счет охвата многих районов и городов республики и объективным ростом заболеваемости паращитовидных желез в Российской Федерации в целом.

В ходе исследования установлено, что на территории Чувашской Республики с численностью населения 1 167 061 человек зарегистрировано 856 случаев заболеваний паращитовидных желез, что соответствует уровню 73,4 случая на 100 000 населения.

Распределение по годам внесения записей в электронный регистр позволяет провести анализ: в 2023 г. было зарегистрировано 10 человек (1,2%), в 2024 г. — 529 человек (61,8%), а в 2025 г. — 317 человек (37%). Средний возраст пациентов составляет 59 лет, что свидетельствует о преобладании лиц старшей возрастной категории. Возрастная структура распределяется следующим образом: наибольшую группу составляют пациенты старше 60 лет (56,4%), затем следуют лица в возрасте 45–60 лет (28,9%), и младше 45 лет (14,7%).

Особого внимания заслуживает гендерное распределение: соотношение женщин и мужчин составляет 5,4:1, что указывает на значительное преобладание женского контингента в регистре.

Анализ географического распределения случаев заболеваний паращитовидных желез в Чувашской Республике выявил неравномерность распространения патологии (табл. 1). Наибольшее абсолютное число случаев зарегистрировано в г. Чебоксары (421 случай; 49,2%) и г. Новочебоксарск (93 случая; 10,9%), что соответствует концентрации распределения лечебно-профилактических учреждений и доступности современных диагностических технологий. При расчете на численность населения наиболее высокие показатели распространенности отмечаются в Чебоксарском районе (0,12%), Канашском (0,1%) и Батыревском (0,1%) муниципальных округах. Отдельно следует отметить 73 случая без указания адреса практического проживания, что требует уточнения данных для совершенствования системы учета.

**Table:** Таблица 1. Распределение пациентов с заболеваниями паращитовидных желез в Чувашской Республике по районам проживания

Место проживания	Количество пациентов	Процентное соотношение на численность населения района (%)
г. Чебоксары	421	0,08
г. Новочебоксарск	93	0,07
Канашский м. о.	33	0,1
Батыревский м. о.	33	0,1
Чебоксарский район	27	0,12
Шумерлинский м. о.	26	0,08
Цивильский м. о.	24	0,08
Алатырский м. о.	20	0,04
Урмарский м. о.	13	0,06
Моргаушский м. о.	11	0,03
Козловский м. о.	10	0,07
Комсомольский м. о.	10	0,05
Вурнарский м. о.	8	0,03
Ибресинский м. о.	7	0,03
Красночетайский м. о.	7	0,05
Мариинско-Посадский м. о.	7	0,04
Порецкий м. о.	6	0,06
Янтиковский м. о.	6	0,04
Яльчикский м. о.	6	0,04
Ядринский м. о.	4	0,02
Аликовский м. о.	4	0,03
Шемуршинский м. о.	4	0,03
Без указания адреса	73	-

По состоянию на сентябрь 2025 г. в БУ ЧР «Республиканский клинический госпиталь для ветеранов войн» Минздрава Чувашии под диспансерным наблюдением с установленным диагнозом первичного гиперпаратиреоза состоит когорта из 550 пациентов. Распределение пациентов по полу (11,1% мужчин и 88,9% женщин) в исследованной выборке отражает известную предрасположенность к ПГПТ среди лиц женского пола, особенно в постменопаузальном периоде.

Наблюдается значительный рост общей обращаемости с 2018 г. (38 пациентов) до пиковых значений в 2024 (353 пациента) и 2025 (223 пациента) гг. (табл. 2). Это может свидетельствовать как о повышении выявляемости эндокринных патологий, так и о расширении охвата медицинской помощью.В начальный период (2014–2018 гг.) процент оперативных вмешательств был высоким и крайне вариабельным (от 15,9 до 40%), что, вероятно, связано с малым абсолютным числом пациентов (n=38–104). Резкое снижение доли оперативного лечения в 2019–2021 гг. до 0,7–5,3% было обусловлено ограничением плановой хирургической помощи в связи с пандемией COVID-19, что совпало с периодом роста общей обращаемости. Начиная с 2022 г., отмечается рост абсолютного числа оперированных пациентов (с n=5 до n=43) в связи с открытием хирургического отделения, профилирующегося на лечении патологии эндокринной системы. Значимый прирост количества выполненных паратиреоидэктомий связан также с выстроенной системой преемственности в оказании медицинской помощи. Созданные клинико-организационные связи с ФГБУ «Федеральный центр травматологии, ортопедии и эндопротезирования»Минздрава России (г. Чебоксары) и БУЧР «Республиканская клиническая больница» Министерства здравоохранения Чувашской Республики, а также функционирование в структуре госпиталя центра профилактики мочекаменной болезни способствуют активному выявлению и направлению пациентов с осложненными формами ПГПТ для хирургического лечения. Это говорит о становлении стабильной и отлаженной системы отбора пациентов на хирургическое лечение, которая функционирует на фоне высокой общей нагрузки. Хирургическая тактика определялась характером поражения: при солитарной аденоме с верифицированной топической диагностикой выполнялась селективная паратиреоидэктомия; при множественном поражении паращитовидных желез проводилась билатеральная ревизия с последующим удалением всех патологически измененных желез.

**Table:** Таблица 2. Анализ обращаемости пациентов к врачу-эндокринологу и процент оперативных вмешательств за 2014–2025 гг. в БУ ЧР «Республиканский клинический госпиталь для ветеранов войн» Минздрава Чувашии

Год	Количество пациентов, обратившихся к врачу-эндокринологу	Оперативное лечение, %
2025	223	11,2 (n=25)
2024	353	12,1 (n=43)
2023	315	13 (n=41)
2022	201	2,5 (n=5)
2021	132	0,7 (n=1)
2020	178	3,4 (n=6)
2019	113	5,3 (n=6)
2018	38	21 (n=8)
2017	63	15,9 (n=10)
2016	104	12,5 (n=13)
2015	77	10,4 (n=8)
2014	40	40 (n=4)

За период с 2022 по 2024 гг. доля впервые в жизни установленных диагнозов первичного гиперпаратиреоза (ПГПТ) в исследуемой когорте составила 5%.

После анализа всех обследованных пациентов с 2014 по 2024 гг. была сделана разбивка пациентов на следующие группы: с первичным гиперпаратиреозом (ПГПТ) со пределенной топической диагностикой и требующих хирургического вмешательства — 170 человек, вторая группа с первичным гиперпаратиреозом, которым требовалось наблюдение и консервативное лечение у эндокринолога — 88 человек, третья группа со вторичным гиперпаратиреозом (ВГПТ), которым требовалось наблюдение и консервативное лечение у эндокринолога — 81 человек, четвертая группа пациентов с гиперпаратиреозом (ГПТ) — 163 человека, которым требовалось дообследование для определения дальнейшей тактики лечения.

Среди 170 прооперированных пациентов большинство (152 человека, или 89,4%) составили женщины, в то время как на долю мужчин пришлось 18 случаев (10,6%). Было выделено две группы в зависимости от уровня кальция в крови, гиперкальциемическая форма преобладает по распространенности над нормокальциемической формой ПГПТ (табл. 3).

**Table:** Таблица 3. Распределение прооперированных пациентов с ПГПТ по полу и клинической форме в БУ ЧР «Республиканский клинический госпиталь для ветеранов войн» Минздрава Чувашии за 2014–2025 гг.

Пол	Количество пациентов с гиперкальциемической формой ПГПТ	Количество пациентов с нормокальциемической формой ПГПТ	Количество пациентов
Женский	107	45	152
Мужской	13	5	18

По результатам исследования среди осложнений первичного гиперпаратиреоза классические органные проявления были представлены остеопорозом, верифицированным у 38,2% (n=65) пациентов, низкотравматическими переломами — 18,2% (n=31), нефролитиазом, диагностированным у 16,5% (n=28), конкрементами мочевого пузыря 0,6% (n=1). Особое внимание привлекает распространенность кардиоваскулярных осложнений, которая достигла 40% (n=68), в их структуре абсолютно преобладала артериальная гипертензия (94,1%, n=64), также отмечались ишемическая болезнь сердца (n=10), гипертрофия левого желудочка (n=3), а также единичные случаи кальцификации сосудов и клапанов сердца, острого инфаркта миокарда и атриовентрикулярной блокады (n=1).

Структура послеоперационных осложнений представлена следующимобразом: кровотечение из послеоперационной раны в первые сутки послеудаления околощитовидных желез — у 1 пациента (0,6%), с целью остановки кровотечения была выполнена ревизия раны, рецидивов не наблюдалось. Парез возвратного гортанного нерва наблюдался у одногопациента (0,6%). Среди жалоб пациенты отмечали дисфонию и затрудненное дыхание прифизической нагрузке. За больными было установлено динамическое наблюдение, через 3–6 месяцев полное восстановление функции возвратного гортанного нерва и отсутствие жалоб. Гипопаратиреоз обнаружен у 3 пациентов (1,8%), их беспокоили судороги и мышечные спазмы. Синдром «голодных костей» выявлен у 2 пациентов (1,2%), больные жаловались на боль в костях и суставах, быструю утомляемость, слабость.

Зарегистрировано 7 (4,1%) случаев рецидива гиперпаратиреоза. В структуре рецидивов в 2 случаях заболевание рецидивировало в течение первого года после операции, а в 5 случаях была выявлена персистенция заболевания, обусловленная наличием аденомы контралатеральной паращитовидной железы. При анализе случаев рецидива ПГПТ скрининг на МЭН1-ассоциированный гиперпаратиреоз не выполнялся, решение обусловлено отсутствием клинических предпосылок для данного синдрома. Ни у одного из пациентов не было зафиксировано указаний на многоочаговый характер поражения паращитовидных желез во время первичной операции, сопутствующих нейроэндокринных опухолей или поражения гипофиза, а также отягощенного семейного анамнеза в отношении МЭН1. Кроме того, возрастная структура данной подгруппы не соответствовала типичному для МЭН1 молодому возрасту манифестации.

Гистологический анализ операционного материала у пациентов подтвердил наличие доброкачественных аденом паращитовидных желез. Структура гистологических вариантов была следующей: аденомы, состоящие из главных клеток, выявлены в 91случае (53,5%), из светлых клеток — в 29 случаях (17,2%), смешанные формы — в 21случае (12,1%), гиперплазия паращитовидных желёз — в 29 случаях (17,2%).

## Обсуждение

По данным исследования Мокрышевой Н.Г., Мирной С.С., Добревой Е.А. и др. отмечается рост выявляемости ПГПТ в РФ по сравнению с 2016 г., что связано с активным началом работы по регистрации пациентов в регионах [1]. Общая численность пациентов с ПГПТ в регистре на 31.12.2017 г. составила 1914 человек (0,001% населения РФ), средний возраст пациентов на момент установки диагноза составил 55,6±10 лет. Из-за отсутствия полномасштабных данных по эпидемиологии заболеваний околощитовидных желез (первичного гиперпаратиреоза и гипопаратиреоза) разработка и ведение соответствующих медицинских регистров с возможностью удаленного сбора информации из разных регионов являются крайне перспективным направлением. Эта инициатива обладает высокой практической и научной ценностью.

Авторы отмечают высокую концентрацию зарегистрированных случаев заболеваний паращитовидных желез в Чебоксарах и Новочебоксарске, которая объясняется группами факторов. В Чебоксарах расположен единственный в республике Республиканский эндокринологический диспансер, куда направляются пациенты со всей Чувашии для диагностики, хирургического лечения и динамического наблюдения, что приводит к концентрации в столице зарегистрированных случаев. Кроме того, в крупных городах выше доступность современных лабораторных исследований: в Чебоксарах и Новочебоксарске активно проводится определение уровня паратгормона и кальция в крови, в то время как в районах определение кальция часто не входит в рутинный биохимический анализ, что приводит к поздней диагностике.

Результаты ранее проведенных исследований свидетельствуют о наличии значительного дисбаланса микроэлементов в водных источниках Чувашской Республики, что может выступать дополнительным фактором риска развития первичного гиперпаратиреоза [14]. Наиболее выраженные нарушения минерального состава водных ресурсов зафиксированы в урбанизированных территориях (г. Чебоксары, г. Новочебоксарск) и отдельных муниципальных образованиях (Батыревский, Канашский, Чебоксарский районы), что формирует зоны повышенного экологического риска для населения. Ключевым механизмом развития заболевания является избыточное содержание лития в воде, который снижает чувствительность кальций-чувствительных рецепторов паращитовидных желез, приводя к компенсаторной повышенной секреции паратгормона. Дополнительными факторами выступают дисбаланс фтора и бора, нарушающих нормальный обмен кальция в организме. Значительный вклад в экологическое неблагополучие вносят промышленные предприятия Чебоксар и Новочебоксарска, осуществляющие выбросы хлора, формальдегида и тяжелых металлов. Эти вещества обладают доказанным повреждающим действием на эндокринную систему и могут способствовать развитию патологии паращитовидных желез.

Данная структура распространения позволяет сделать вывод о наибольшей охваченности медицинским учетом городского населения республики, в то время как в районах сохраняется необходимость усиления работы по выявляемости и регистрации пациентов.

Данные по Тюменской области демонстрируют сходную с нашей когортой возрастную структуру (средний возраст 60±11,05 года) и выраженное преобладание женщин (94,0%) среди пациентов с ПГПТ [15]. Ключевым отличием является более высокая доля манифестных форм (80,1%), что может указывать на более позднюю диагностику в регионе, а также на потенциальные различия в диагностических подходах. Особого внимания заслуживает высокая частота кардиоваскулярных осложнений (артериальная гипертензия — 86,9%, ИБС, ГЛЖ — 68,1%), что подтверждает системное влияние ПГПТ и подчеркивает необходимость включения оценки кальциевого обмена в алгоритм обследования пациентов с сердечно-сосудистой патологией. Выявленный повсеместный дефицит витамина D (у 68% обследованных) согласуется с общероссийскими тенденциями и является значимым модифицируемым фактором, требующим коррекции.

Проведенный анализ в БУ ЧР «Республиканский клинический госпиталь для ветеранов войн» Минздрава Чувашии демонстрирует значительный рост выявляемости первичного гиперпаратиреоза (ПГПТ) в Чувашской Республике, что, вероятно, связано с улучшением диагностических возможностей и повышением настороженности врачей в отношении данной патологии. Выявленное гендерное распределение (11,1% мужчин и 88,9% женщин) полностью соответствует общемировым эпидемиологическим данным о значительном преобладании ПГПТ среди женщин, особенно в постменопаузальном периоде. Низкий процент (5%) впервые установленных диагнозов в общей когорте за последние три года косвенно указывает на накопление значительного контингента пациентов с длительно текущим заболеванием, что требует дальнейшего изучения отдаленных исходов и оптимизации системы диспансерного наблюдения.

## Заключение

Внедрение Всероссийского регистра пациентов с первичным гиперпаратиреозом. В Чувашской Республике позволило впервые получить эпидемиологические данные. Установленная распространенность патологии составила 73,4 случая на 100 000 населения на 2025 г.

Выявлена неравномерность географического распределения пациентов с максимальной концентрацией случаев в г. Чебоксары и г. Новочебоксарск, что объясняется доступностью специализированной помощи, возможным влиянием неблагоприятных экологических факторов, включая дисбаланс микроэлементов (лития, бора) в водных источниках.

Анализ динамики обращаемости в БУ ЧР «Республиканский клинический госпиталь для ветеранов войн» Минздрава Чувашии выявил значительный рост выявляемости первичного гиперпаратиреоза (ПГПТ) в период с 2018 по 2024 гг., что свидетельствует об улучшении диагностических возможностей и расширении охвата медицинской помощью.

Регистр доказал свою практическую ценность как инструмент для мониторинга заболеваемости, планирования хирургической помощи и проведения научных исследований, однако требует дальнейшего развития для улучшения охвата и уточнения данных, особенно в районах республики.

## ДОПОЛНИТЕЛЬНАЯ ИНФОРМАЦИЯ

Источник финансирования. Работа выполнена по инициативе авторов без привлечения финансирования.

Конфликт интересов. Авторы сообщают, что не имеют финансовых, личных или профессиональных интересов, которые могли бы повлиять на данное исследование.

Вклад авторов. Все авторы одобрили финальную версию статьи перед публикацией, выразили согласие нести ответственность за все аспекты работы, подразумевающую надлежащее изучение ирешение вопросов, связанных с точностью или добросовестностью любой части работы.
